# 2045 The role of TGFβ in driving early cystic fibrosis lung disease

**DOI:** 10.1017/cts.2018.139

**Published:** 2018-11-21

**Authors:** Elizabeth L. Kramer, William Hardie, Kristin Hudock, Cynthia Davidson, Alicia Ostmann, John P. Clancy

**Affiliations:** Cincinnati Children’s Hospital Medical Center

## Abstract

OBJECTIVES/SPECIFIC AIMS: Transforming growth factor-beta (TGFβ) is a genetic modifier of cystic fibrosis (CF) lung disease. TGFβ’s pulmonary levels in young CF patients and its mechanism of action in CF are unknown. We examined TGFβ levels in children with CF and investigated responses of human airway epithelial cells (AECs) and mice to TGFβ. METHODS/STUDY POPULATION: TGFβ levels in bronchoalveolar lavage fluid from CF patients (n=15) and non-CF control patients (n=21)<6 years old were determined by ELISA. CF mice and non-CF mice were intratracheally treated with an adenoviral TGFβ1 vector or PBS; lungs were collected for analysis at day 7. Human CF and non-CF AECs were treated with TGFβ or PBS for 24 hours then collected for analysis. RESULTS/ANTICIPATED RESULTS: Young CF patients had higher bronchoalveolar lavage fluid TGFβ than non-CF controls (*p*=0.03). Mouse lungs exposed to TGFβ demonstrated inflammation, goblet cell hyperplasia, and decreased CFTR expression. CF mice had greater TGFβ-induced lung mechanics abnormalities than controls; both CF human AECs and CF mice showed higher TGFβ induced MAPK and PI3K signaling compared with controls. DISCUSSION/SIGNIFICANCE OF IMPACT: For the first time, we show increased TGFβ levels very early in CF. TGFβ drives CF lung abnormalities in mouse and human models; CF models are more sensitive to TGFβ’s effects. Understanding the role of TGFβ in promoting CF lung disease is critical to developing patient specific treatments.

